# Crystal structure and Hirshfeld surface analysis of 2-benzyl-4,5-di­bromo-2,3,3a,4,5,6,7,7a-octa­hydro-3a,6-ep­oxy-1*H*-isoindol-1-one

**DOI:** 10.1107/S2056989021001481

**Published:** 2021-02-12

**Authors:** Dmitriy F. Mertsalov, Vladimir P. Zaytsev, Kuzma M. Pokazeev, Mikhail S. Grigoriev, Alexander V. Bachinsky, Sevim Türktekin Çelikesir, Mehmet Akkurt, Sixberth Mlowe

**Affiliations:** a Department of Organic Chemistry, Peoples’ Friendship University of Russia (RUDN University), 6 Miklukho-Maklaya St., 117198, Moscow, Russian Federation; b Frumkin Institute of Physical Chemistry and Electrochemistry, Russian Academy of Sciences, Leninsky pr. 31, bld. 4, Moscow, 119071, Russian Federation; c Department of Physics, Faculty of Sciences, Erciyes University, 38039 Kayseri, Turkey; d University of Dar es Salaam, Dar es Salaam University College of Education, Department of Chemistry, PO Box 2329, Dar es Salaam, Tanzania

**Keywords:** crystal structure, pyrrolidine ring, tetra­hydro­furan ring, ep­oxy­iso­indole moiety, Hirshfeld surface analysis

## Abstract

The title compound, C_15_H_15_Br_2_NO_2_, crystallizes with two mol­ecules in the asymmetric unit of the unit cell. In both mol­ecules, the tetra­hydro­furan rings adopt an envelope conformation with the O atom as the flap and the pyrrolidine rings adopt an envelope conformation. In the crystal, mol­ecules are linked by weak C—H⋯O hydrogen bonds, forming sheets lying parallel to the (002) plane. These sheets are connected only by weak van der Waals inter­actions. The most important contributions to the surface contacts are from H⋯H (44.6%), Br⋯H/H⋯Br (24.1%), O⋯H/H⋯O (13.5%) and C⋯H/H⋯C (11.2%) inter­actions, as concluded from a Hirshfeld surface analysis.

## Abstract 2

In the crystal, mol­ecules are linked by weak C—H⋯O hydrogen bonds, forming sheets lying parallel to the (002) plane. These sheets are connected only by weak van der Waals inter­actions.

## Chemical context   

**Figure 1:**
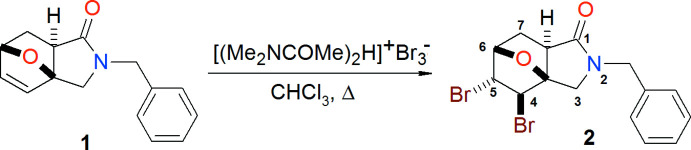
Synthesis scheme for 2-benzyl-4,5-di­bromo­hexa­hydro-3a,6-ep­oxy­isoindol-1(4*H*)-one (**2**)

Halogenation is a chemical reaction that involves the introduction of one or more halogen atoms to an organic mol­ecule. The pathway and stereochemistry of halogenation reactions is dependent on the configuration of the starting olefine and the halogenating agent. The role/behavior of the attached halogen atom in olefines can be classified into the following types: (1) as an electron-withdrawing substituent, (2) as a halogen-bond donor center, and (3) as a non-covalent bond acceptor site. Thus, not only hydrogen bonding (Gurbanov *et al.*, 2018 ▸; Kopylovich *et al.*, 2011 ▸) or other types of non-covalent inter­actions (Afkhami *et al.*, 2017 ▸; Asadov *et al.*, 2016 ▸; Ma *et al.*, 2017*a* ▸,*b* ▸; 2020 ▸; Mahmudov *et al.*, 2010 ▸; 2019 ▸; 2020 ▸, but also halogen bonding can be used in the design of olefines. In this work, we proposed and tested inexpensive and readily available bis­[*N*,*N*-di­methyl­acetamide] hydrogen di­bromo­bromate [(Me_2_NCOMe)_2_H]Br_3_ as a bromine initiator and a source of a positively charged bromine ion (Rodygin *et al.*, 1992 ▸; Prokop’eva *et al.*, 1994 ▸; Prokop’eva, 2008 ▸). The choice of [(Me_2_NCOMe)_2_H]Br_3_, obtained by one-pot synthesis from *N*,*N*-di­methyl­acetamide, hydro­bromic acid and bromine, is down to the simplicity of the synthesis and isolation, and the unambiguous direction of the bromination process. In addition, [(Me_2_NCOMe)_2_H]Br_3_ is an excellent reagent for functionalization of phenols and anilines (Rodygin *et al.*, 1992 ▸; Mikhailov *et al.*, 1993 ▸), and is also used in the synthesis of mono-bromo-substituted ketones (Rodygin *et al.*, 1994 ▸; Burakov *et al.*, 2001 ▸) and for the bromination of various alkenes and alkynes (Rodygin *et al.*, 1994 ▸; Zaytsev *et al.*, 2017 ▸). The present work is aimed at accumulating experimental data and establishing the rules of the halogenation in bridged ep­oxy-isoindolones (Zubkov *et al.*, 2018 ▸; Zaytsev *et al.*, 2020 ▸). The reaction of *N*-benzyl­tetra­hydro­epoxy­isoindolone (**1**) with [(Me_2_NCOMe)_2_H]Br_3_ in dry chloro­form under reflux leads to the corresponding 2-benzyl-4,5-di­bromo­hexa­hydro-3a,6-ep­oxy­isoindol-1(4*H*)-one (**2**) (Fig. 1 ▸).
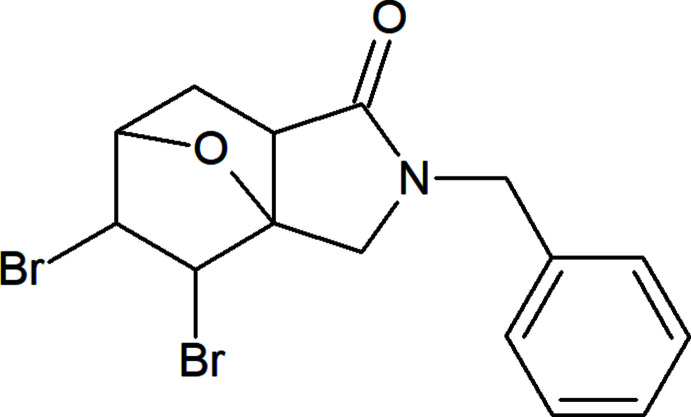


## Structural commentary   

**Figure 2:**
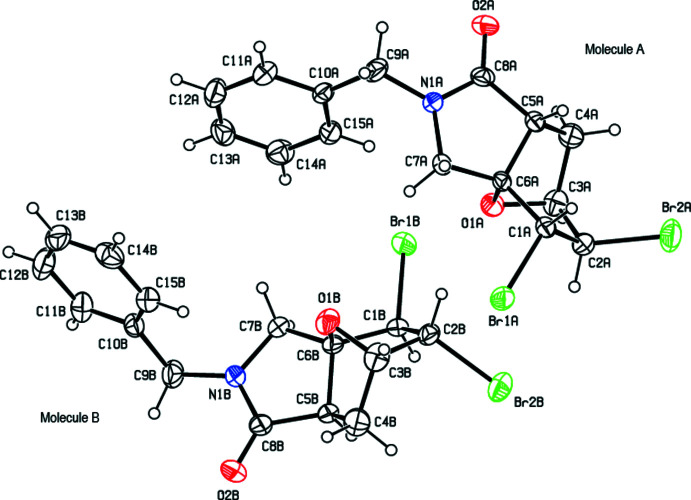
The two mol­ecules (*A* and *B*) in the asymmetric unit of the title compound with displacement ellipsoids for the non-hydrogen atoms drawn at the 30% probability level.

**Figure 3:**
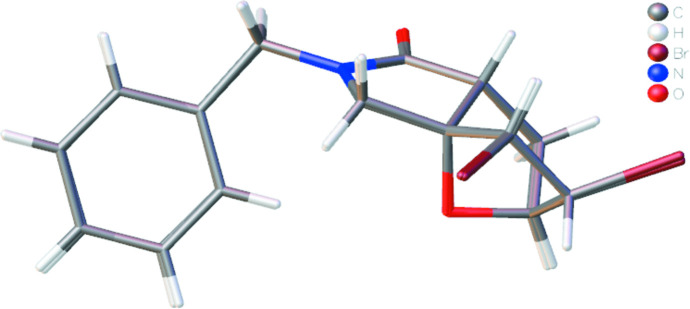
Overlay image (*OLEX2*; Dolomanov *et al.*, 2009 ▸) of the two mol­ecules (*A* and *B*) in the asymmetric unit of the title compound.

The asymmetric unit of the title compound (Fig. 2 ▸) contains two mol­ecules of similar shape, hereafter referred to as mol­ecules *A* (including atom C1*A*) and *B* (including atom C1*B*). The conformational differences between mol­ecules *A* and *B* are highlighted in an overlay diagram shown in Fig. 3 ▸. The r.m.s. deviation of the overlay between the mol­ecules *A* and *B* is 0.114 Å.

In both mol­ecules *A* and *B*, the pyrrolidine rings (N1*A*/C5*A*–C8*A* and N1*B*/C5*B*–C8*B*), tetra­hydro­furan rings (O1*A*/C1*A*–C3*A*/C6*A*, O1*A*/C3*A*–C6*A* and O1*B*/C1*B*–C3*B*/C6*B*, O1*B*/C3*B*–C6*B*) and six-membered rings (C1*A*–C6*A* and C1*B*–C6*B*), which generate ep­oxy­iso­indole moieties (O1*A*/N1*A*/C1*A*–C8*A* and O1*B*/N1*B*/C1*B*–C8*B*), are puckered. In mol­ecule *A*, both tetra­hydro­furan rings adopt an envelope conformation with puckering parameters (Cremer & Pople, 1975 ▸) *Q*(2) = 0.575 (3) Å, φ(2) = 182.2 (4)° for (O1*A*/C1*A*–C3*A*/C6*A*) and *Q*(2) = 0.558 (4) Å, φ(2) = 3.8 (4)° for (O1*A*/C3*A*–C6*A*), respectively. In mol­ecule *B*, both tetra­hydro­furan rings also adopt an envelope conformation with puckering parameters *Q*(2) = 0.575 (4) Å, φ(2) = 182.7 (4)° for (O1*B*/C1*B*–C3*B*/C6*B*) and *Q*(2) = 0.556 (4) Å, φ(2) = 3.7 (4)° for (O1*B*/C3*B*–C6*B*).

The five-membered pyrrolidine rings also exhibit an envelope conformation, with a maximum deviation from the mean plane of 0.155 (3) Å at C6*A* [puckering parameters *Q*(2) = 0.248 (4) Å, φ(2) = 77.7 (8)°] for mol­ecule *A* and 0.153 (3) Å at C6*B* [puckering parameters *Q*(2) = 0.243 (4) Å, φ(2) = 75.0 (9)°] for mol­ecule *B*. In both mol­ecules, the six-membered ring has a boat conformation [*Q*_T_ = 0.921 (4) Å, θ = 91.8 (2)°, φ = 119.3 (2)° for mol­ecule *A*; *Q*_T_ = 0.919 (4) Å, θ = 91.9 (2)°, φ = 119.6 (3)° for mol­ecule *B*].

## Supra­molecular features   

**Figure 4:**
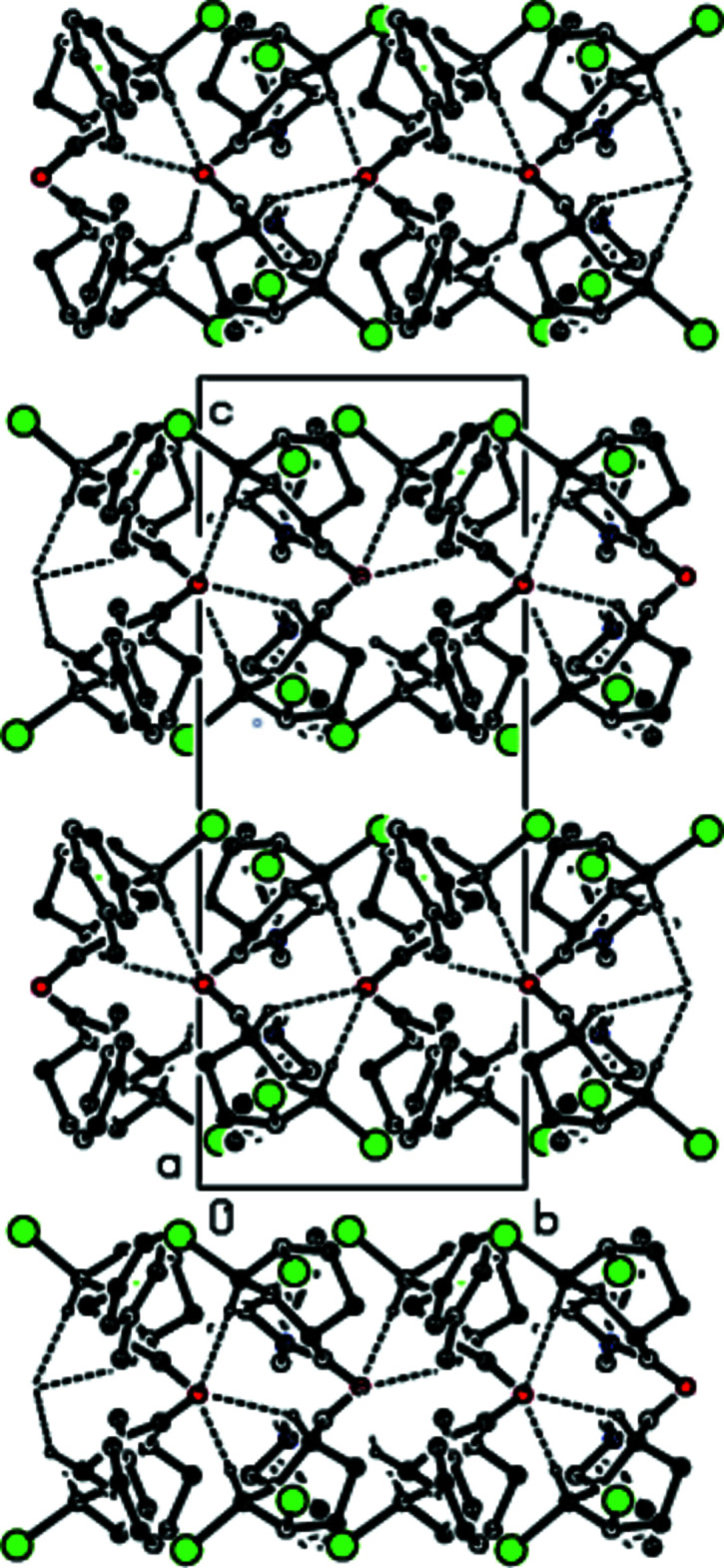
A view along the *a* axis of the inter­molecular C—H⋯O inter­actions in the title compound.

In the crystal, mol­ecules are linked by weak C—H⋯O hydrogen bonds, forming sheets lying parallel to the (002) plane (Table 1 ▸, Figs. 4 ▸ and 5 ▸). These sheets are connected only by weak van der Waals inter­actions.

## Hirshfeld surface analysis   

**Figure 6:**
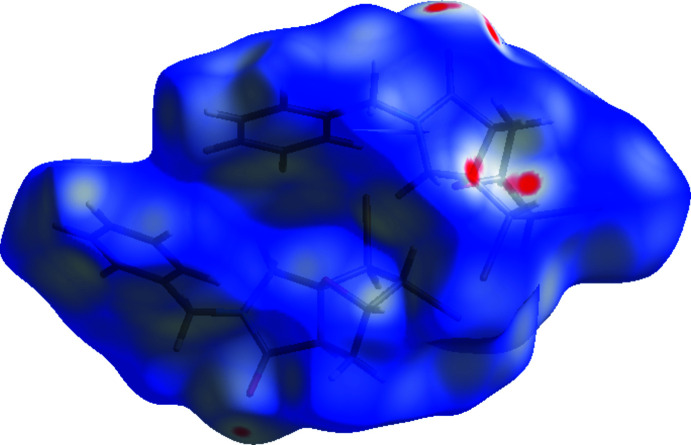
A view of the three-dimensional Hirshfeld surface for the title compound, plotted over *d*_norm_ in the range −0.0815 to 0.9926 a.u.

**Figure 7:**
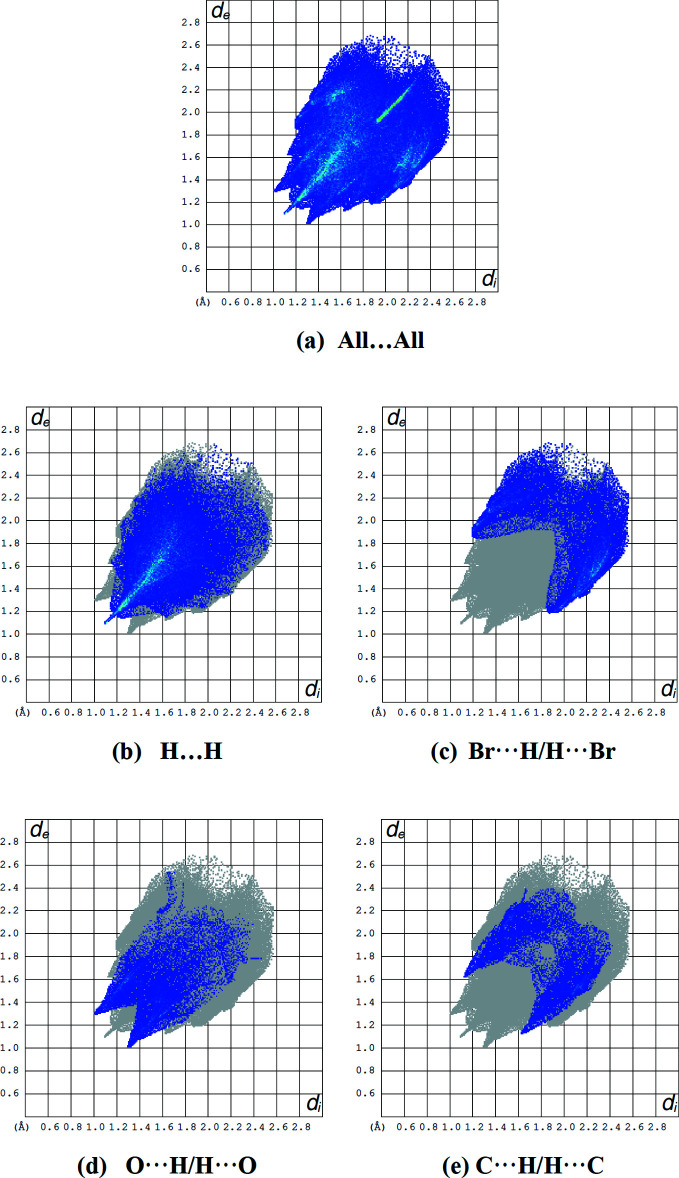
A view of the two-dimensional fingerprint plots for the title compound, showing (*a*) all inter­actions, and delineated into (*b*) H⋯H, (*c*) Br⋯H/H⋯Br, (*d*) O⋯H/H⋯O and (*e*) C⋯H/H⋯C inter­actions. The *d*_i_ and *d*_e_ values are the closest inter­nal and external distances (in Å) from given points on the Hirshfeld surface.

The inter­molecular inter­actions (Table 2 ▸) were investigated qu­anti­tatively and visualized with *Crystal Explorer 3.1* (Wolff *et al.*, 2012 ▸; Spackman *et al.*, 2009 ▸). The Hirshfeld surface plotted over *d*_norm_ in the range −0.0815 to 0.9926 a.u. is shown in Fig. 6 ▸. The red spots on the Hirshfeld surface represent C—H⋯O contacts. Fig. 7 ▸ shows the full two-dimensional fingerprint plot and those delineated into the major contacts: the H⋯H (44.6%) inter­actions are the major factor in the crystal packing with Br⋯H/H⋯Br (24.1%), O⋯H/H⋯O (13.5%) and C⋯H/H⋯C (11.2%) inter­actions representing the next highest contributions. The percentage contributions of other weak inter­actions are listed in Table 3 ▸.

## Database survey   

A search of the Cambridge Structural Database (CSD version 5.40, update of September 2019; Groom *et al.*, 2016 ▸) for structures having the ep­oxy­iso­indole moiety gave six hits, which closely resemble the title compound, *viz*. (3a*R*,6*S*,7a*R*)-7a-chloro-2-[(4-nitro­phen­yl)sulfon­yl]-1,2,3,6,7,7a-hexa­hydro-3a,6-ep­oxy­iso­indole (CSD refcode AGONUH; Temel *et al.*, 2013 ▸), (3a*R*,6*S*,7a*R*)-7a-chloro-6-methyl-2-[(4-nitro­phen­yl)sulfon­yl]-1,2,3,6,7,7a-hexa­hydro-3a,6-ep­oxy­iso­indole (TIJ­MIK; Demircan *et al.*, 2013 ▸), 5-chloro-7-methyl-3-[(4-methyl­phen­yl)sulfon­yl]-10-oxa-3-azatri­cyclo­[5.2.1.01,5]dec-8-ene (YAXCIL; Temel *et al.*, 2012 ▸), (3a*R*,6*S*,7a*R*)-7a-bromo-2-[(4-methyl­phen­yl)sulfon­yl]-1,2,3,6,7,7a-hexa­hydro-3a,6-ep­oxy­iso­indole (UPAQEI; Koşar *et al.*, 2011 ▸), (3a*R*,6*S*,7a*R*)-7a-bromo-2-methyl­sulfonyl-1,2,3,6,7,7a-hexa­hydro-3a,6-ep­oxy­iso­indole (ERIVIL; Temel *et al.*, 2011 ▸) and *tert*-butyl 3a-chloro­per­hydro-2,6a-ep­oxy­oxireno(*e*)iso­indole-5-carboxyl­ate (MIG­TIG; Koşar *et al.*, 2007 ▸).

In the crystal of AGONUH, the mol­ecules are linked by C—H⋯O hydrogen bonds into zigzag chains running along the *b*-axis direction. In TIJMIK, two types of C—H⋯O hydrogen bonds generate 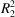
(20) and 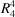
(26) rings, with adjacent rings running parallel to *ac* plane. In addition C—H⋯O hydrogen bonds form a *C*(6) chain, linking the mol­ecules in the *b*-axis direction. In YAXCIL and UPAQEI, mol­ecules are also linked by C—H⋯O hydrogen bonds. In the crystal of ERIVIL, weak inter­molecular C—H⋯O hydrogen bonds link the mol­ecules into 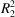
(8) and 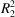
(14) rings along the *b*-axis direction. In MIGTIG, the mol­ecules are linked only by weak van der Waals inter­actions.

## Synthesis and crystallization   

A solution of isoindolone **1** (4 mmol) and the brominating agent (4 mmol) in dry chloro­form (15 mL) was heated under reflux for 18 h (TLC control, EtOAc–hexane, 1:1). The reaction mixture was poured into H_2_O (50 mL) and extracted with CHCl_3_ (3 × 20 mL). The combined organic fractions were dried over anhydrous Na_2_SO_4_, the solvent was evaporated under reduced pressure, and the residue was purified by column chromatography (SiO_2_, 15 × 1.8 cm, hexa­ne/EtOAc, 10:1). Colourless hexa­gonal prisms. Yield 0.46 g (29%), m.p. > 428 K (decomposition).

IR (KBr), ν (cm^−1^): 1693 (N—C=O), 627 (C—Br). ^1^H NMR (CDCl_3_, 600 MHz, 301 K): δ = 7.37–7.34 (*m*, 2H, H-Ph), 7.32–7.29 (*m*, 1H, H-Ph), 7.24–7.22 (*m*, 2H, H-Ph), 4.71 (*t*, 1H, H6, *J* = 5.0), 4.54 (*d*, 1H, CH_2_Ph, *J* = 15.1) and 4.48 (*d*, 1H, CH_2_Ph, *J* = 15.1), 4.46 (*ddd*, 1H, H5, *J* = 1.5, *J* = 2.5, *J* = 5.0), 4.17 (*d*, 1H, H4, *J* = 2.5), 3.50 (*d*, 1H, *J* = 12.1) and 3.47 (*d*, 1H, H3, *J* = 12.1), 2.81 (*dd*, 1H, H7*A*, *J* = 4.7, J = 9.3), 2.73 (*dd*, 1H, H7*B*, *J* = 9.3, *J* = 12.8), 2.25–2.21 (*m*, 1H, H7*A*). ^13^C NMR (CDCl_3_, 150.9 MHz, 301 K): *δ* = 172.9, 135.5, 128.8 (2C), 127.9 (2C), 127.7, 90.0, 80.6, 55.1, 48.9, 48.4, 46.5, 33.8, 30.2. MS (APCI): *m*/*z* = 400 [*M* + H]^+^ (^81^Br), 402 [*M* + H]^+^ (^81^Br, ^79^Br), 404 [*M* + H]^+^ (^79^Br).

## Refinement   

Crystal data, data collection and structure refinement details are summarized in Table 4 ▸. C-bound H atoms were positioned geometrically, with C—H = 0.93 Å (for aromatic H atoms), C—H = 0.98 Å (for methine H atoms), 0.97 Å (for methyl­ene H atoms) and 0.96 Å (for methyl H atoms), and constrained to ride on their parent atoms, with *U*_iso_(H) =1.2*U*_eq_(C) or 1.5*U*_eq_(C-meth­yl). Ten reflections (101), (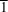
01), (111), (002), (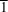
11), (110), (200), (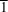
03), (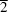
02) and (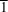
12) were obscured by the beam stop and omitted during the final refinement cycle.

## Supplementary Material

Crystal structure: contains datablock(s) I, global. DOI: 10.1107/S2056989021001481/jy2005sup1.cif

Structure factors: contains datablock(s) I. DOI: 10.1107/S2056989021001481/jy2005Isup2.hkl

CCDC reference: 2061968

Additional supporting information:  crystallographic information; 3D view; checkCIF report

## Figures and Tables

**Figure 5:**
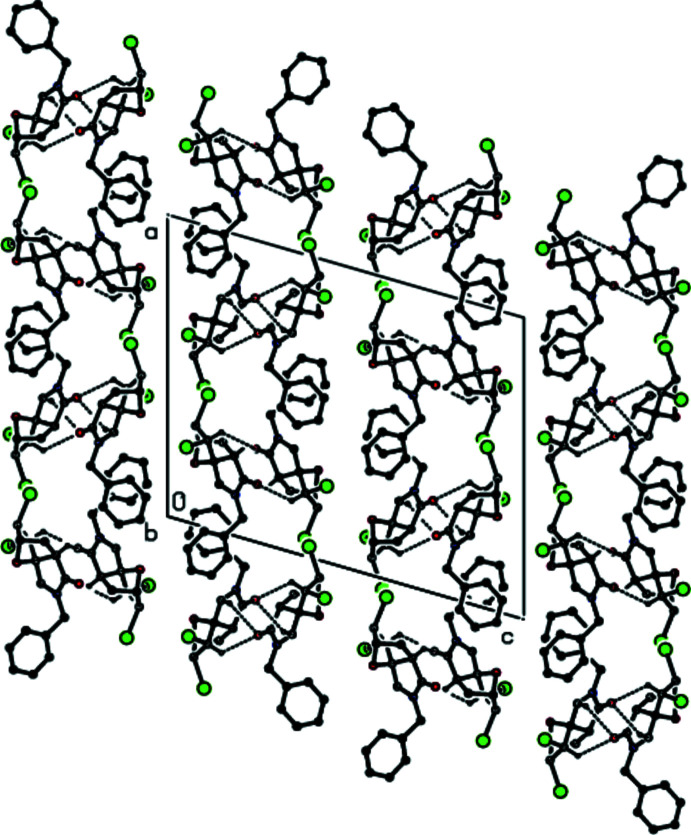
A view along the *b* axis of the inter­molecular C—H⋯O inter­actions in the title compound.

**Table 1:** Hydrogen-bond geometry (Å, °)

*D*—H⋯*A*	*D*—H	H⋯*A*	*D*⋯*A*	*D*—H⋯*A*
C1*A*—H1*A*⋯O2*A* ^i^	0.98	2.51	3.175 (4)	125
C4*A*—H4*AB*⋯Br2*A*	0.97	2.81	3.287 (4)	111
C5*A*—H5*A*⋯O2*A* ^i^	0.98	2.64	3.271 (4)	123
C7*A*—H7*AB*⋯O2*A* ^i^	0.97	2.53	3.186 (4)	125
C1*B*—H1*B*⋯O2*B* ^ii^	0.98	2.39	3.104 (4)	129
C2*B*—H2*B*⋯O1*A*	0.98	2.38	3.341 (4)	168
C4*B*—H4*BB*⋯Br2*B*	0.97	2.83	3.301 (4)	111
C5*B*—H5*B*⋯O2*B* ^ii^	0.98	2.57	3.247 (5)	126
C7*B*—H7*BB*⋯O2*B* ^ii^	0.97	2.64	3.270 (5)	123

Symmetry codes: (i) -x+{\script{3\over 2}}, y+{\script{1\over 2}}, -z+{\script{1\over 2}}; (ii) -x+{\script{3\over 2}}, y-{\script{1\over 2}}, -z+{\script{3\over 2}}.

**Table 2:** Summary of short inter­atomic contacts (Å) in the title compound

Contact	Distance	Symmetry operation
H2*A*⋯Br1*A*	3.21	2 − *x*, 1 − *y*, 1 − *z*
Br1*A*⋯Br2*B*	3.8655	2 − *x*, 1 − *y*, 1 − *z*
Br2*A*⋯Br1*B*	3.8993	2 − *x*, −*y*, 1 − *z*
O1*A*⋯H2*B*	2.38	*x*, *y*, *z*
O2*A*⋯H1*A*	2.51	{3\over 2} − *x*, −{1\over 2} + *y*, {1\over 2} − *z*
O2*A*⋯H11*B*	2.78	1 − *x*, − *y*, 1 − *z*
H7*AA*⋯C13*B*	2.85	1 − *x*, 1 − *y*, 1 − *z*
H11*A*⋯H5*B*	2.55	−{1\over 2} + *x*, {1\over 2} − *y*, −{1\over 2} + *z*
H13*B*⋯Br1*B*	3.13	1 − *x*, −*y*, 1 − *z*
O2*B*⋯H1*B*	2.39	{3\over 2} − *x*, {1\over 2} + *y*, {3\over 2} − *z*
H13*B*⋯H3*B*	2.42	1 − *x*, 1 − *y*, 1 − *z*

**Table 3:** Percentage contributions of inter­atomic contacts to the Hirshfeld surface for the title compound

Contact	Percentage contribution
H⋯H	44.6
Br⋯H/H⋯Br	24.1
O⋯H/H⋯O	13.5
C⋯H/H⋯C	11.2
Br⋯Br	3.9
C⋯C	2.0
N⋯H/H⋯N	0.5
Br⋯C/C⋯Br	0.3

**Table 4:** Experimental details

Crystal data	
Chemical formula	C_15_H_15_Br_2_NO_2_
*M*_r_	401.10
Crystal system, space group	Monoclinic, *P*2_1_/*n*
Temperature (K)	296
*a*, *b*, *c* (Å)	17.4839 (5), 8.2993 (3), 21.5120 (7)
β (°)	106.115 (2)
*V* (Å^3^)	2998.83 (17)
*Z*	8
Radiation type	Mo *K*α
μ (mm^−1^)	5.41
Crystal size (mm)	0.14 × 0.13 × 0.13

Data collection	
Diffractometer	Bruker Kappa APEXII area-detector
Absorption correction	Multi-scan (*SADABS*; Bruker, 2008 ▸)
*T*_min_, *T*_max_	0.224, 0.294
No. of measured, independent and observed [*I* > 2σ(*I*)] reflections	32806, 6995, 3997
*R*_int_	0.053
(sin θ/λ)_max_ (Å^−1^)	0.657

Refinement	
*R*[*F* ^2^ > 2σ(*F* ^2^)], *wR*(*F* ^2^), *S*	0.043, 0.091, 1.02
No. of reflections	6995
No. of parameters	362
H-atom treatment	H-atom parameters constrained
Δρ_max_, Δρ_min_ (e Å^−3^)	0.83, −0.66

Computer programs: *APEX2* (Bruker, 2013 ▸), *SAINT* (Bruker, 2013 ▸), *SHELXT* (Sheldrick, 2015*a* ▸), *SHELXL* (Sheldrick, 2015*b* ▸), *ORTEP-3 for Windows* (Farrugia, 2012 ▸), *OLEX2* (Dolomanov *et al.*, 2009 ▸) and *PLATON* (Spek, 2020 ▸).

## supplementary crystallographic information

### Crystal data

**Table:**

C_15_H_15_Br_2_NO_2_	*F*(000) = 1584
*M**_r_* = 401.10	*D*_x_ = 1.777 Mg m^−^^3^
Monoclinic, *P*2_1_/*n*	Mo *K*α radiation, λ = 0.71073 Å
*a* = 17.4839 (5) Å	Cell parameters from 5179 reflections
*b* = 8.2993 (3) Å	θ = 2.7–22.8°
*c* = 21.5120 (7) Å	µ = 5.41 mm^−^^1^
β = 106.115 (2)°	*T* = 296 K
*V* = 2998.83 (17) Å^3^	Hexagonal prisms, colourless
*Z* = 8	0.14 × 0.13 × 0.13 mm

### Data collection

**Table:**

Bruker Kappa APEXII area-detector diffractometer	3997 reflections with *I* > 2σ(*I*)
ω– and φ–scans	*R*_int_ = 0.053
Absorption correction: multi-scan (SADABS; Bruker, 2008)	θ_max_ = 27.8°, θ_min_ = 2.7°
*T*_min_ = 0.224, *T*_max_ = 0.294	*h* = −22→22
32806 measured reflections	*k* = −10→10
6995 independent reflections	*l* = −28→27

### Refinement

**Table:**

Refinement on *F*^2^	Hydrogen site location: inferred from neighbouring sites
Least-squares matrix: full	H-atom parameters constrained
*R*[*F*^2^ > 2σ(*F*^2^)] = 0.043	*w* = 1/[σ^2^(*F*_o_^2^) + (0.0333*P*)^2^ + 1.282*P*] where *P* = (*F*_o_^2^ + 2*F*_c_^2^)/3
*wR*(*F*^2^) = 0.091	(Δ/σ)_max_ = 0.001
*S* = 1.02	Δρ_max_ = 0.83 e Å^−^^3^
6995 reflections	Δρ_min_ = −0.65 e Å^−^^3^
362 parameters	Extinction correction: SHELXL2018/3 (Sheldrick 2015b), Fc^*^=kFc[1+0.001xFc^2^λ^3^/sin(2θ)]^-1/4^
0 restraints	Extinction coefficient: 0.00111 (12)
Primary atom site location: inferred from neighbouring sites	

### Special details

**Table:**

Geometry. All esds (except the esd in the dihedral angle between two l.s. planes) are estimated using the full covariance matrix. The cell esds are taken into account individually in the estimation of esds in distances, angles and torsion angles; correlations between esds in cell parameters are only used when they are defined by crystal symmetry. An approximate (isotropic) treatment of cell esds is used for estimating esds involving l.s. planes.

### Fractional atomic coordinates and isotropic or equivalent isotropic displacement parameters (Å^2^)

**Table:**

	*x*	*y*	*z*	*U*_iso_*/*U*_eq_	
C1A	0.8852 (2)	0.3745 (4)	0.38724 (16)	0.0370 (9)	
H1A	0.900973	0.406568	0.348719	0.044*	
C2A	0.9420 (2)	0.2461 (5)	0.42647 (17)	0.0449 (10)	
H2A	0.959259	0.280858	0.471765	0.054*	
C3A	0.8874 (2)	0.0992 (5)	0.42168 (18)	0.0482 (10)	
H3A	0.907172	0.020343	0.456328	0.058*	
C4A	0.8625 (2)	0.0245 (5)	0.35457 (19)	0.0519 (11)	
H4AA	0.833459	−0.075272	0.354065	0.062*	
H4AB	0.907972	0.004926	0.338033	0.062*	
C5A	0.8091 (2)	0.1574 (4)	0.31692 (16)	0.0362 (9)	
H5A	0.831483	0.203675	0.283872	0.043*	
C6A	0.80785 (19)	0.2812 (4)	0.36906 (15)	0.0309 (8)	
C7A	0.72741 (19)	0.3587 (4)	0.34938 (16)	0.0373 (9)	
H7AA	0.707948	0.380283	0.386626	0.045*	
H7AB	0.728396	0.458539	0.326150	0.045*	
C8A	0.7213 (2)	0.1203 (5)	0.28926 (16)	0.0413 (9)	
C9A	0.5932 (2)	0.2502 (5)	0.28436 (17)	0.0511 (11)	
H9AA	0.574028	0.171921	0.250087	0.061*	
H9AB	0.579798	0.356414	0.265650	0.061*	
C10A	0.5498 (2)	0.2245 (4)	0.33480 (17)	0.0381 (9)	
C11A	0.4700 (2)	0.2679 (5)	0.31950 (19)	0.0495 (10)	
H11A	0.445253	0.311234	0.279045	0.059*	
C12A	0.4276 (2)	0.2472 (5)	0.3638 (2)	0.0607 (12)	
H12A	0.374225	0.276410	0.353302	0.073*	
C13A	0.4637 (3)	0.1837 (5)	0.4234 (2)	0.0606 (12)	
H13A	0.434686	0.168605	0.453196	0.073*	
C14A	0.5425 (3)	0.1423 (5)	0.4392 (2)	0.0573 (11)	
H14A	0.567212	0.100717	0.479959	0.069*	
C15A	0.5855 (2)	0.1619 (5)	0.39485 (17)	0.0454 (10)	
H15A	0.638886	0.132644	0.405694	0.055*	
N1A	0.67922 (17)	0.2373 (4)	0.30754 (13)	0.0413 (8)	
O1A	0.81551 (14)	0.1790 (3)	0.42508 (10)	0.0424 (6)	
O2A	0.69344 (17)	0.0056 (3)	0.25485 (13)	0.0602 (8)	
Br1A	0.87859 (2)	0.56161 (5)	0.44107 (2)	0.05631 (15)	
Br2A	1.03562 (3)	0.20912 (6)	0.39717 (3)	0.07454 (18)	
C1B	0.7908 (2)	0.1347 (4)	0.61250 (16)	0.0372 (9)	
H1B	0.829984	0.094825	0.651364	0.045*	
C2B	0.8272 (2)	0.2662 (5)	0.57905 (17)	0.0440 (10)	
H2B	0.815399	0.240013	0.532932	0.053*	
C3B	0.7797 (2)	0.4175 (5)	0.58646 (19)	0.0520 (11)	
H3B	0.780526	0.501711	0.554716	0.062*	
C4B	0.7985 (3)	0.4797 (5)	0.6555 (2)	0.0576 (12)	
H4BA	0.772113	0.581506	0.657849	0.069*	
H4BB	0.855305	0.491715	0.674785	0.069*	
C5B	0.7640 (2)	0.3436 (4)	0.68693 (16)	0.0379 (9)	
H5B	0.806381	0.287933	0.719270	0.045*	
C6B	0.7267 (2)	0.2315 (4)	0.63022 (15)	0.0342 (8)	
C7B	0.6560 (2)	0.1563 (5)	0.64522 (17)	0.0435 (9)	
H7BA	0.612042	0.144230	0.606419	0.052*	
H7BB	0.669218	0.051786	0.665507	0.052*	
C8B	0.6947 (2)	0.3797 (5)	0.71379 (18)	0.0454 (10)	
C9B	0.5654 (2)	0.2577 (6)	0.71072 (19)	0.0575 (12)	
H9BA	0.568277	0.335108	0.745061	0.069*	
H9BB	0.564186	0.150991	0.728853	0.069*	
C10B	0.4888 (2)	0.2840 (4)	0.65875 (18)	0.0419 (9)	
C11B	0.4196 (2)	0.2234 (5)	0.6684 (2)	0.0561 (11)	
H11B	0.421680	0.166181	0.705987	0.067*	
C12B	0.3474 (3)	0.2465 (6)	0.6231 (3)	0.0698 (14)	
H12B	0.301036	0.205823	0.630294	0.084*	
C13B	0.3441 (3)	0.3291 (6)	0.5677 (3)	0.0691 (14)	
H13B	0.295513	0.343200	0.536779	0.083*	
C14B	0.4122 (3)	0.3918 (5)	0.5572 (2)	0.0618 (12)	
H14B	0.409600	0.449397	0.519547	0.074*	
C15B	0.4852 (2)	0.3686 (5)	0.60312 (19)	0.0509 (10)	
H15B	0.531509	0.410312	0.596131	0.061*	
N1B	0.63674 (18)	0.2718 (4)	0.68980 (14)	0.0465 (8)	
O1B	0.70287 (14)	0.3452 (3)	0.57735 (11)	0.0503 (7)	
O2B	0.69187 (17)	0.4868 (4)	0.75178 (14)	0.0648 (8)	
Br1B	0.74753 (3)	−0.04142 (6)	0.55333 (2)	0.06418 (16)	
Br2B	0.94187 (2)	0.28604 (6)	0.61425 (2)	0.06177 (15)	

### Atomic displacement parameters (Å^2^)

**Table:**

	*U*^11^	*U*^22^	*U*^33^	*U*^12^	*U*^13^	*U*^23^
C1A	0.037 (2)	0.040 (2)	0.036 (2)	−0.0011 (18)	0.0147 (17)	−0.0043 (17)
C2A	0.038 (2)	0.055 (3)	0.039 (2)	0.0064 (19)	0.0054 (18)	−0.0034 (19)
C3A	0.046 (2)	0.048 (2)	0.048 (2)	0.008 (2)	0.008 (2)	0.013 (2)
C4A	0.052 (3)	0.039 (2)	0.065 (3)	−0.002 (2)	0.017 (2)	−0.010 (2)
C5A	0.037 (2)	0.039 (2)	0.035 (2)	−0.0019 (17)	0.0145 (17)	−0.0052 (17)
C6A	0.0317 (19)	0.037 (2)	0.0262 (18)	0.0007 (17)	0.0116 (15)	0.0010 (16)
C7A	0.034 (2)	0.043 (2)	0.038 (2)	−0.0003 (18)	0.0151 (17)	−0.0061 (18)
C8A	0.047 (2)	0.051 (3)	0.027 (2)	−0.010 (2)	0.0127 (18)	−0.0056 (19)
C9A	0.034 (2)	0.074 (3)	0.039 (2)	−0.003 (2)	0.0010 (18)	0.004 (2)
C10A	0.031 (2)	0.042 (2)	0.039 (2)	−0.0070 (17)	0.0072 (17)	−0.0031 (18)
C11A	0.038 (2)	0.056 (3)	0.051 (2)	−0.004 (2)	0.005 (2)	−0.001 (2)
C12A	0.038 (2)	0.059 (3)	0.088 (4)	−0.004 (2)	0.022 (3)	−0.009 (3)
C13A	0.061 (3)	0.065 (3)	0.068 (3)	−0.016 (3)	0.038 (3)	−0.014 (3)
C14A	0.061 (3)	0.066 (3)	0.045 (3)	−0.011 (2)	0.015 (2)	0.002 (2)
C15A	0.038 (2)	0.055 (3)	0.043 (2)	−0.0004 (19)	0.0104 (19)	0.0004 (19)
N1A	0.0313 (16)	0.058 (2)	0.0347 (17)	−0.0048 (15)	0.0094 (14)	−0.0095 (15)
O1A	0.0450 (15)	0.0525 (16)	0.0315 (14)	−0.0023 (13)	0.0140 (12)	0.0063 (12)
O2A	0.0576 (19)	0.071 (2)	0.0518 (17)	−0.0166 (16)	0.0157 (15)	−0.0287 (15)
Br1A	0.0431 (2)	0.0517 (3)	0.0735 (3)	−0.0083 (2)	0.0150 (2)	−0.0237 (2)
Br2A	0.0425 (3)	0.0729 (3)	0.1129 (4)	0.0071 (2)	0.0293 (3)	−0.0161 (3)
C1B	0.034 (2)	0.045 (2)	0.0293 (19)	−0.0019 (18)	0.0033 (16)	−0.0069 (17)
C2B	0.033 (2)	0.066 (3)	0.032 (2)	−0.0049 (19)	0.0068 (17)	−0.0002 (19)
C3B	0.049 (3)	0.055 (3)	0.052 (3)	0.004 (2)	0.014 (2)	0.019 (2)
C4B	0.057 (3)	0.039 (2)	0.079 (3)	−0.003 (2)	0.021 (2)	−0.007 (2)
C5B	0.0310 (19)	0.045 (2)	0.034 (2)	0.0007 (17)	0.0029 (16)	−0.0066 (17)
C6B	0.0319 (19)	0.041 (2)	0.0261 (19)	0.0007 (17)	0.0029 (15)	0.0007 (16)
C7B	0.031 (2)	0.054 (3)	0.042 (2)	−0.0040 (18)	0.0058 (17)	−0.0103 (19)
C8B	0.039 (2)	0.059 (3)	0.034 (2)	0.008 (2)	0.0017 (18)	−0.004 (2)
C9B	0.041 (2)	0.088 (3)	0.048 (2)	0.003 (2)	0.019 (2)	0.005 (2)
C10B	0.038 (2)	0.041 (2)	0.049 (2)	0.0043 (19)	0.0166 (19)	−0.0047 (19)
C11B	0.046 (3)	0.051 (3)	0.077 (3)	0.005 (2)	0.027 (2)	0.004 (2)
C12B	0.038 (3)	0.062 (3)	0.112 (4)	−0.002 (2)	0.024 (3)	−0.010 (3)
C13B	0.043 (3)	0.068 (3)	0.085 (4)	0.016 (2)	−0.001 (3)	−0.026 (3)
C14B	0.076 (3)	0.054 (3)	0.053 (3)	0.017 (3)	0.014 (3)	−0.005 (2)
C15B	0.046 (3)	0.057 (3)	0.051 (3)	−0.002 (2)	0.016 (2)	−0.004 (2)
N1B	0.0353 (18)	0.069 (2)	0.0368 (18)	−0.0002 (17)	0.0119 (15)	−0.0119 (17)
O1B	0.0382 (15)	0.0691 (19)	0.0386 (15)	0.0098 (14)	0.0022 (12)	0.0128 (13)
O2B	0.0585 (19)	0.078 (2)	0.0557 (18)	0.0063 (16)	0.0126 (15)	−0.0312 (16)
Br1B	0.0532 (3)	0.0681 (3)	0.0736 (3)	−0.0076 (2)	0.0215 (2)	−0.0344 (2)
Br2B	0.0391 (2)	0.0748 (3)	0.0719 (3)	−0.0073 (2)	0.0161 (2)	0.0053 (2)

### Geometric parameters (Å, º)

**Table:**

C1A—C6A	1.513 (5)	C1B—C6B	1.512 (5)
C1A—C2A	1.539 (5)	C1B—C2B	1.539 (5)
C1A—Br1A	1.959 (3)	C1B—Br1B	1.948 (3)
C1A—H1A	0.9800	C1B—H1B	0.9800
C2A—C3A	1.534 (5)	C2B—C3B	1.538 (5)
C2A—Br2A	1.935 (4)	C2B—Br2B	1.943 (3)
C2A—H2A	0.9800	C2B—H2B	0.9800
C3A—O1A	1.440 (4)	C3B—O1B	1.434 (4)
C3A—C4A	1.520 (5)	C3B—C4B	1.520 (5)
C3A—H3A	0.9800	C3B—H3B	0.9800
C4A—C5A	1.525 (5)	C4B—C5B	1.524 (5)
C4A—H4AA	0.9700	C4B—H4BA	0.9700
C4A—H4AB	0.9700	C4B—H4BB	0.9700
C5A—C8A	1.516 (5)	C5B—C8B	1.510 (5)
C5A—C6A	1.526 (4)	C5B—C6B	1.529 (4)
C5A—H5A	0.9800	C5B—H5B	0.9800
C6A—O1A	1.449 (4)	C6B—O1B	1.447 (4)
C6A—C7A	1.497 (4)	C6B—C7B	1.497 (5)
C7A—N1A	1.455 (4)	C7B—N1B	1.459 (4)
C7A—H7AA	0.9700	C7B—H7BA	0.9700
C7A—H7AB	0.9700	C7B—H7BB	0.9700
C8A—O2A	1.221 (4)	C8B—O2B	1.218 (4)
C8A—N1A	1.341 (5)	C8B—N1B	1.343 (5)
C9A—N1A	1.452 (4)	C9B—N1B	1.443 (4)
C9A—C10A	1.501 (5)	C9B—C10B	1.504 (5)
C9A—H9AA	0.9700	C9B—H9BA	0.9700
C9A—H9AB	0.9700	C9B—H9BB	0.9700
C10A—C15A	1.371 (5)	C10B—C15B	1.374 (5)
C10A—C11A	1.389 (5)	C10B—C11B	1.378 (5)
C11A—C12A	1.371 (5)	C11B—C12B	1.377 (6)
C11A—H11A	0.9300	C11B—H11B	0.9300
C12A—C13A	1.368 (6)	C12B—C13B	1.362 (6)
C12A—H12A	0.9300	C12B—H12B	0.9300
C13A—C14A	1.369 (6)	C13B—C14B	1.374 (6)
C13A—H13A	0.9300	C13B—H13B	0.9300
C14A—C15A	1.377 (5)	C14B—C15B	1.394 (5)
C14A—H14A	0.9300	C14B—H14B	0.9300
C15A—H15A	0.9300	C15B—H15B	0.9300
			
C6A—C1A—C2A	100.4 (3)	C6B—C1B—C2B	100.0 (3)
C6A—C1A—Br1A	111.3 (2)	C6B—C1B—Br1B	112.5 (2)
C2A—C1A—Br1A	111.1 (2)	C2B—C1B—Br1B	111.3 (2)
C6A—C1A—H1A	111.2	C6B—C1B—H1B	110.9
C2A—C1A—H1A	111.2	C2B—C1B—H1B	110.9
Br1A—C1A—H1A	111.2	Br1B—C1B—H1B	110.9
C3A—C2A—C1A	102.6 (3)	C3B—C2B—C1B	103.0 (3)
C3A—C2A—Br2A	114.8 (3)	C3B—C2B—Br2B	114.8 (3)
C1A—C2A—Br2A	114.0 (2)	C1B—C2B—Br2B	113.2 (2)
C3A—C2A—H2A	108.4	C3B—C2B—H2B	108.5
C1A—C2A—H2A	108.4	C1B—C2B—H2B	108.5
Br2A—C2A—H2A	108.4	Br2B—C2B—H2B	108.5
O1A—C3A—C4A	102.3 (3)	O1B—C3B—C4B	102.4 (3)
O1A—C3A—C2A	99.6 (3)	O1B—C3B—C2B	99.0 (3)
C4A—C3A—C2A	113.4 (3)	C4B—C3B—C2B	113.7 (3)
O1A—C3A—H3A	113.4	O1B—C3B—H3B	113.4
C4A—C3A—H3A	113.4	C4B—C3B—H3B	113.4
C2A—C3A—H3A	113.4	C2B—C3B—H3B	113.4
C3A—C4A—C5A	100.4 (3)	C3B—C4B—C5B	100.2 (3)
C3A—C4A—H4AA	111.7	C3B—C4B—H4BA	111.7
C5A—C4A—H4AA	111.7	C5B—C4B—H4BA	111.7
C3A—C4A—H4AB	111.7	C3B—C4B—H4BB	111.7
C5A—C4A—H4AB	111.7	C5B—C4B—H4BB	111.7
H4AA—C4A—H4AB	109.5	H4BA—C4B—H4BB	109.5
C8A—C5A—C4A	117.8 (3)	C8B—C5B—C4B	118.7 (3)
C8A—C5A—C6A	101.9 (3)	C8B—C5B—C6B	102.4 (3)
C4A—C5A—C6A	103.2 (3)	C4B—C5B—C6B	103.3 (3)
C8A—C5A—H5A	111.1	C8B—C5B—H5B	110.5
C4A—C5A—H5A	111.1	C4B—C5B—H5B	110.5
C6A—C5A—H5A	111.1	C6B—C5B—H5B	110.5
O1A—C6A—C7A	110.5 (3)	O1B—C6B—C7B	111.5 (3)
O1A—C6A—C1A	102.4 (3)	O1B—C6B—C1B	102.7 (3)
C7A—C6A—C1A	123.7 (3)	C7B—C6B—C1B	123.2 (3)
O1A—C6A—C5A	101.6 (3)	O1B—C6B—C5B	101.3 (3)
C7A—C6A—C5A	106.6 (3)	C7B—C6B—C5B	106.1 (3)
C1A—C6A—C5A	109.9 (3)	C1B—C6B—C5B	110.0 (3)
N1A—C7A—C6A	102.4 (3)	N1B—C7B—C6B	102.9 (3)
N1A—C7A—H7AA	111.3	N1B—C7B—H7BA	111.2
C6A—C7A—H7AA	111.3	C6B—C7B—H7BA	111.2
N1A—C7A—H7AB	111.3	N1B—C7B—H7BB	111.2
C6A—C7A—H7AB	111.3	C6B—C7B—H7BB	111.2
H7AA—C7A—H7AB	109.2	H7BA—C7B—H7BB	109.1
O2A—C8A—N1A	125.7 (4)	O2B—C8B—N1B	125.3 (4)
O2A—C8A—C5A	125.9 (3)	O2B—C8B—C5B	126.0 (4)
N1A—C8A—C5A	108.4 (3)	N1B—C8B—C5B	108.7 (3)
N1A—C9A—C10A	115.1 (3)	N1B—C9B—C10B	115.1 (3)
N1A—C9A—H9AA	108.5	N1B—C9B—H9BA	108.5
C10A—C9A—H9AA	108.5	C10B—C9B—H9BA	108.5
N1A—C9A—H9AB	108.5	N1B—C9B—H9BB	108.5
C10A—C9A—H9AB	108.5	C10B—C9B—H9BB	108.5
H9AA—C9A—H9AB	107.5	H9BA—C9B—H9BB	107.5
C15A—C10A—C11A	119.0 (3)	C15B—C10B—C11B	119.2 (4)
C15A—C10A—C9A	123.1 (3)	C15B—C10B—C9B	122.5 (3)
C11A—C10A—C9A	117.9 (3)	C11B—C10B—C9B	118.3 (4)
C12A—C11A—C10A	120.3 (4)	C12B—C11B—C10B	120.9 (4)
C12A—C11A—H11A	119.9	C12B—C11B—H11B	119.6
C10A—C11A—H11A	119.9	C10B—C11B—H11B	119.6
C13A—C12A—C11A	120.2 (4)	C13B—C12B—C11B	119.8 (4)
C13A—C12A—H12A	119.9	C13B—C12B—H12B	120.1
C11A—C12A—H12A	119.9	C11B—C12B—H12B	120.1
C12A—C13A—C14A	120.0 (4)	C12B—C13B—C14B	120.5 (4)
C12A—C13A—H13A	120.0	C12B—C13B—H13B	119.8
C14A—C13A—H13A	120.0	C14B—C13B—H13B	119.8
C13A—C14A—C15A	120.2 (4)	C13B—C14B—C15B	119.7 (4)
C13A—C14A—H14A	119.9	C13B—C14B—H14B	120.2
C15A—C14A—H14A	119.9	C15B—C14B—H14B	120.2
C10A—C15A—C14A	120.3 (4)	C10B—C15B—C14B	120.0 (4)
C10A—C15A—H15A	119.8	C10B—C15B—H15B	120.0
C14A—C15A—H15A	119.8	C14B—C15B—H15B	120.0
C8A—N1A—C9A	123.6 (3)	C8B—N1B—C9B	124.0 (3)
C8A—N1A—C7A	114.4 (3)	C8B—N1B—C7B	113.8 (3)
C9A—N1A—C7A	121.9 (3)	C9B—N1B—C7B	121.9 (3)
C3A—O1A—C6A	96.2 (2)	C3B—O1B—C6B	96.4 (2)
			
C6A—C1A—C2A—C3A	2.0 (3)	C6B—C1B—C2B—C3B	2.6 (3)
Br1A—C1A—C2A—C3A	119.8 (3)	Br1B—C1B—C2B—C3B	121.6 (3)
C6A—C1A—C2A—Br2A	126.7 (2)	C6B—C1B—C2B—Br2B	127.1 (2)
Br1A—C1A—C2A—Br2A	−115.4 (2)	Br1B—C1B—C2B—Br2B	−113.9 (2)
C1A—C2A—C3A—O1A	−37.2 (3)	C1B—C2B—C3B—O1B	−37.7 (3)
Br2A—C2A—C3A—O1A	−161.4 (2)	Br2B—C2B—C3B—O1B	−161.2 (2)
C1A—C2A—C3A—C4A	70.7 (4)	C1B—C2B—C3B—C4B	70.2 (4)
Br2A—C2A—C3A—C4A	−53.5 (4)	Br2B—C2B—C3B—C4B	−53.2 (4)
O1A—C3A—C4A—C5A	37.9 (3)	O1B—C3B—C4B—C5B	37.9 (4)
C2A—C3A—C4A—C5A	−68.4 (4)	C2B—C3B—C4B—C5B	−67.9 (4)
C3A—C4A—C5A—C8A	−114.6 (3)	C3B—C4B—C5B—C8B	−116.0 (3)
C3A—C4A—C5A—C6A	−3.4 (4)	C3B—C4B—C5B—C6B	−3.5 (4)
C2A—C1A—C6A—O1A	33.9 (3)	C2B—C1B—C6B—O1B	33.4 (3)
Br1A—C1A—C6A—O1A	−83.8 (3)	Br1B—C1B—C6B—O1B	−84.7 (3)
C2A—C1A—C6A—C7A	159.2 (3)	C2B—C1B—C6B—C7B	160.0 (3)
Br1A—C1A—C6A—C7A	41.5 (4)	Br1B—C1B—C6B—C7B	41.9 (4)
C2A—C1A—C6A—C5A	−73.4 (3)	C2B—C1B—C6B—C5B	−73.8 (3)
Br1A—C1A—C6A—C5A	168.9 (2)	Br1B—C1B—C6B—C5B	168.1 (2)
C8A—C5A—C6A—O1A	91.0 (3)	C8B—C5B—C6B—O1B	92.6 (3)
C4A—C5A—C6A—O1A	−31.6 (3)	C4B—C5B—C6B—O1B	−31.4 (3)
C8A—C5A—C6A—C7A	−24.7 (3)	C8B—C5B—C6B—C7B	−24.0 (4)
C4A—C5A—C6A—C7A	−147.3 (3)	C4B—C5B—C6B—C7B	−147.9 (3)
C8A—C5A—C6A—C1A	−161.1 (3)	C8B—C5B—C6B—C1B	−159.3 (3)
C4A—C5A—C6A—C1A	76.3 (3)	C4B—C5B—C6B—C1B	76.8 (3)
O1A—C6A—C7A—N1A	−86.1 (3)	O1B—C6B—C7B—N1B	−86.1 (3)
C1A—C6A—C7A—N1A	152.2 (3)	C1B—C6B—C7B—N1B	151.2 (3)
C5A—C6A—C7A—N1A	23.4 (3)	C5B—C6B—C7B—N1B	23.4 (4)
C4A—C5A—C8A—O2A	−52.9 (5)	C4B—C5B—C8B—O2B	−52.6 (5)
C6A—C5A—C8A—O2A	−164.9 (3)	C6B—C5B—C8B—O2B	−165.6 (4)
C4A—C5A—C8A—N1A	128.8 (3)	C4B—C5B—C8B—N1B	128.6 (4)
C6A—C5A—C8A—N1A	16.8 (4)	C6B—C5B—C8B—N1B	15.6 (4)
N1A—C9A—C10A—C15A	−12.7 (5)	N1B—C9B—C10B—C15B	−22.1 (6)
N1A—C9A—C10A—C11A	166.8 (3)	N1B—C9B—C10B—C11B	159.4 (4)
C15A—C10A—C11A—C12A	−0.5 (6)	C15B—C10B—C11B—C12B	0.1 (6)
C9A—C10A—C11A—C12A	−179.9 (4)	C9B—C10B—C11B—C12B	178.7 (4)
C10A—C11A—C12A—C13A	0.0 (6)	C10B—C11B—C12B—C13B	0.5 (7)
C11A—C12A—C13A—C14A	0.7 (6)	C11B—C12B—C13B—C14B	−1.0 (7)
C12A—C13A—C14A—C15A	−1.0 (6)	C12B—C13B—C14B—C15B	0.9 (6)
C11A—C10A—C15A—C14A	0.1 (6)	C11B—C10B—C15B—C14B	−0.2 (6)
C9A—C10A—C15A—C14A	179.6 (4)	C9B—C10B—C15B—C14B	−178.8 (4)
C13A—C14A—C15A—C10A	0.6 (6)	C13B—C14B—C15B—C10B	−0.3 (6)
O2A—C8A—N1A—C9A	−5.0 (6)	O2B—C8B—N1B—C9B	−6.4 (6)
C5A—C8A—N1A—C9A	173.3 (3)	C5B—C8B—N1B—C9B	172.4 (3)
O2A—C8A—N1A—C7A	179.3 (3)	O2B—C8B—N1B—C7B	−179.9 (4)
C5A—C8A—N1A—C7A	−2.4 (4)	C5B—C8B—N1B—C7B	−1.0 (4)
C10A—C9A—N1A—C8A	113.6 (4)	C10B—C9B—N1B—C8B	119.8 (4)
C10A—C9A—N1A—C7A	−71.0 (4)	C10B—C9B—N1B—C7B	−67.2 (5)
C6A—C7A—N1A—C8A	−13.4 (4)	C6B—C7B—N1B—C8B	−14.3 (4)
C6A—C7A—N1A—C9A	170.8 (3)	C6B—C7B—N1B—C9B	172.0 (3)
C4A—C3A—O1A—C6A	−58.3 (3)	C4B—C3B—O1B—C6B	−58.5 (3)
C2A—C3A—O1A—C6A	58.3 (3)	C2B—C3B—O1B—C6B	58.4 (3)
C7A—C6A—O1A—C3A	167.7 (3)	C7B—C6B—O1B—C3B	167.3 (3)
C1A—C6A—O1A—C3A	−58.7 (3)	C1B—C6B—O1B—C3B	−59.0 (3)
C5A—C6A—O1A—C3A	54.9 (3)	C5B—C6B—O1B—C3B	54.8 (3)

### Hydrogen-bond geometry (Å, º)

**Table:**

*D*—H···*A*	*D*—H	H···*A*	*D*···*A*	*D*—H···*A*
C1*A*—H1*A*···O2*A*^i^	0.98	2.51	3.175 (4)	125
C4*A*—H4*AB*···Br2*A*	0.97	2.81	3.287 (4)	111
C5*A*—H5*A*···O2*A*^i^	0.98	2.64	3.271 (4)	123
C7*A*—H7*AB*···O2*A*^i^	0.97	2.53	3.186 (4)	125
C1*B*—H1*B*···O2*B*^ii^	0.98	2.39	3.104 (4)	129
C2*B*—H2*B*···O1*A*	0.98	2.38	3.341 (4)	168
C4*B*—H4*BB*···Br2*B*	0.97	2.83	3.301 (4)	111
C5*B*—H5*B*···O2*B*^ii^	0.98	2.57	3.247 (5)	126
C7*B*—H7*BB*···O2*B*^ii^	0.97	2.64	3.270 (5)	123

Symmetry codes: (i) −*x*+3/2, *y*+1/2, −*z*+1/2; (ii) −*x*+3/2, *y*−1/2, −*z*+3/2.
